# Genetic analysis reveals the shared genetic architecture between breast cancer and atrial fibrillation

**DOI:** 10.3389/fgene.2025.1450259

**Published:** 2025-03-25

**Authors:** Yang Yang, Jiayi Chen, XiaoHua Zhao, Fuhong Gong, Ruimin Liu, Jingge Miao, Mengping Lin, Fei Ge, Wenlin Chen

**Affiliations:** ^1^ Yunnan Key Laboratory of Breast Cancer Precision Medicine, Department of Breast Surgery, The Third Affiliated Hospital of Kunming Medical University, Peking University Cancer Hospital Yunnan, Yunnan Cancer Hospital, Kunming, China; ^2^ Department of Cardiology, Yan’an Hospital Affiliated To Kunming Medical University, Kunming, China; ^3^ Department of Breast Surgery, First Affiliated Hospital of Kunming Medical University, Kunming, China

**Keywords:** atrial fibrillation, breast cancer, genetic correlation, shared genetic etiology, pleiotropic gene, causal inference

## Abstract

**Background:**

Epidemiological studies have observed an association between atrial fibrillation (AF) and breast cancer (BC). However, the underlying mechanisms linking these two conditions remain unclear. This study aims to systematically explore the genetic association between AF and BC.

**Methods:**

We utilized the largest available genome-wide association study (GWAS) datasets for European individuals, including summary data for AF (N = 1,030,836) and BC (N = 247,173). Multiple approaches were employed to systematically investigate the genetic relationship between AF and BC from the perspectives of pleiotropy and causality.

**Results:**

Global genetic analysis using LDSC and HDL revealed a genetic correlation between AF and BC (rg = 0.0435, P = 0.039). Mixer predicted genetic overlap between non-MHC regions of the two conditions (n = 125, rg = 0.05). Local genetic analyses using LAVA and GWAS-PW identified 22 regions with potential genetic sharing. Cross-trait meta-analysis by CPASSOC identified one novel pleiotropic SNP and 14 pleiotropic SNPs, which were subsequently annotated. Eight of these SNPs passed Bayesian colocalization tests, including one novel pleiotropic SNP. Further fine-mapping analysis identified a set of causal SNPs for each significant SNP. TWAS analyses using JTI and FOCUS models jointly identified 10 pleiotropic genes. Phenome-wide association study (PheWAS) of novel pleiotropic SNPs identified two eQTLs (PELO, ITGA1). Gene-based PheWAS results showed strong associations with BMI, height, and educational attainment. PCGA methods combining GTEx V8 tissue data and single-cell RNA data identified 16 co-enriched tissue types (including cardiovascular, reproductive, and digestive systems) and 5 cell types (including macrophages and smooth muscle cells). Finally, univariable and multivariable bidirectional Mendelian randomization analyses excluded a causal relationship between AF and BC.

**Conclusion:**

This study systematically investigated the shared genetic overlap between AF and BC. Several pleiotropic SNPs and genes were identified, and co-enriched tissue and cell types were revealed. The findings highlight common mechanisms from a genetic perspective rather than a causal relationship. This study provides new insights into the AF-BC association and suggests potential experimental targets and directions for future research. Additionally, the results underscore the importance of monitoring the potential risk of one disease in patients diagnosed with the other.

## Introduction

Cardiovascular diseases and cancer are the two most common diseases that endanger human health (Siegel et al., 2024; Chen et al., 2022; Schnabel et al., 2015). Among them, breast cancer (BC) is one of the main factors endangering women’s health (Siegel et al., 2024), and atrial fibrillation (AF) is one of the most common cardiovascular diseases, also a significant cause of cardiovascular mortality (Chen et al., 2022; Schnabel et al., 2015). Studies have shown that patients with AF are at an increased risk of developing BC (Boutas et al., 2023; Conen et al., 2016; Guzzetti et al., 2008; Yao et al., 2023). Conversely, BC patients are also at an increased risk of cardiovascular diseases (Guha et al., 2022; Matthews et al., 2021; Gulati and Mulvagh, 2018), with AF being the most common among them (D'Souza et al., 2019). Although common factors such as smoking, alcohol consumption, and inflammation partially explain the association between the two diseases (Giza et al., 2017; Koene et al., 2016), the results of studies are contradictory (Guha et al., 2022; Saliba et al., 2018; Wassertheil-Smoller et al., 2017), and the association between BC and AF remains unclear.

Studies have found that the pharmacological treatment of AF is associated with the occurrence of BC (Su et al., 2013). Conversely, the treatment of BC can also increase the incidence of cardiovascular diseases (Gulati and Mulvagh, 2018). However, this still does not explain the potential biological mechanisms between them. Studies have shown that both AF and BC are influenced by genetic factors (Miyazawa et al., 2023; Moller et al., 2016). By using statistical genetics methods to cross-analyze the two diseases, genetic sharing between the two can be discovered (Zhu et al., 2021). This could provide new insights into epidemiological causal inference and potential biological mechanisms.

In this study, we used large-scale Genome-wide association study (GWAS) summary data to delve into the genetic correlations, common risk loci, and potential functions of genes between AF and BC, which may pave the way for precise treatment strategies for patients with AF and BC.

## Methods

### Data sources

We obtained GWAS data containing 60,620 cases of AF and 970,216 controls from a meta-analysis that included six studies (Nielsen et al., 2018). We filtered out single nucleotide polymorphisms (SNPs) with a minor allele frequency of less than 1%. After removing SNPs with duplicate rsids and missing data, we acquired a summary dataset consisting of 9,358,555 autosomal SNPs.

BC GWAS data were sourced from the Breast Cancer Association Consortium (BCAC), which included 133,384 cases and 113,789 controls (Zhang et al., 2020). Similarly, we filtered out SNPs with a minor allele frequency of less than 1% and removed SNPs with duplicate rsids and missing data, ultimately obtaining 9,435,343 autosomal summary data.

**FIGURE 1:**
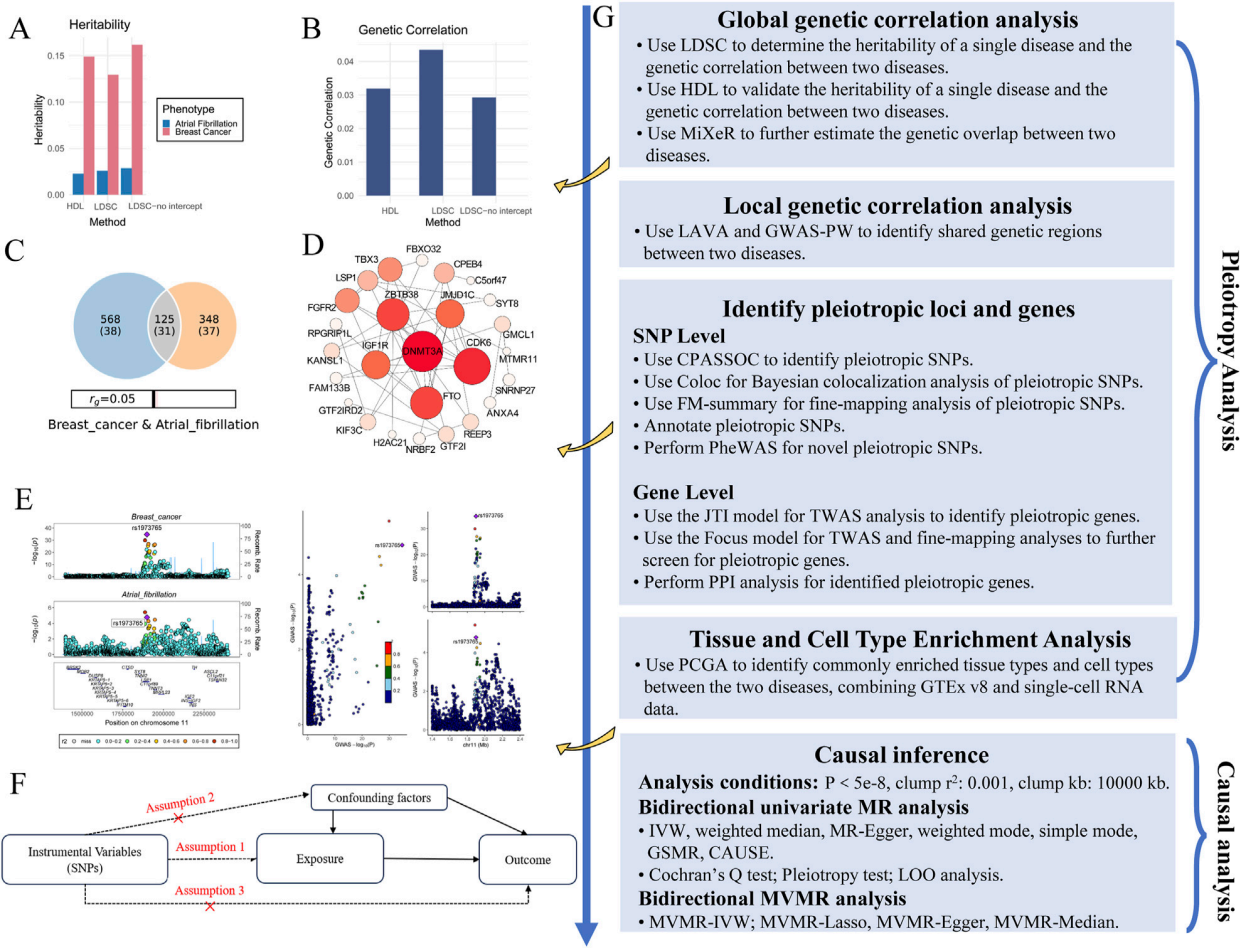
Flowchart of this study. **(A)** Heritability analysis using LDSC for two phenotypes: AF and BC. **(B)** Genetic correlation analysis using LDSC, comparing the genetic correlation between the two diseases. **(C)** Genetic overlap estimated by MiXeR for AF and BC. **(D)** PPI network analysis showing key pleiotropic genes, including DNMT3A, related to both diseases. **(E)** Colocalization analysis of GWAS for both diseases, showing shared genetic variants. **(F)** Basic assumptions in MR analysis, including assumptions related to IVs, exposure, and outcome. **(G)** Flowchart summarizing the genetic and causal analysis steps.

There was no sample overlap between the two GWAS datasets. Both were aligned with the human reference consortium build 37 and originated from European populations (details in Supplementary Table S1). All GWAS datasets were approved by their respective ethical review boards. The overall study process is shown in the figure (Figure 1).

### Global genetic correlation analysis

We performed global genetic correlation analysis using linkage disequilibrium score regression (LDSC) and high-definition likelihood (HDL) methods (Bulik-Sullivan et al., 2015; Ning et al., 2020). The results of genome-wide genetic correlation (rg) ranged from −1 to 1, indicating negative and positive correlations, respectively. LDSC utilized pre-calculated linkage disequilibrium (LD) scores from the 1000 Genomes Project to analyze SNPs in HapMap 3. HDL used reference data from the HapMap3 SNPs imputed version from the United Kingdom Biobank, utilizing full GWAS data for analysis. Compared to LDSC, HDL provides a smaller error estimate for correlation, enhancing result accuracy (Ning et al., 2020).

### Local genetic correlation analysis

We further estimated the local genetic correlation between AF and BC using LAVA and pairwise-GWAS (GWAS-PW) (Pickrell et al., 2016; Werme et al., 2022). GWAS-PW, employing a Bayesian framework, calculated the posterior probabilities of association (PPA) for genomic regions (Pickrell et al., 2016). It used 1,703 pre-partitioned 1000 Genomes Project data as reference. LAVA divided the phase 3 LD panel of the 1,000 Genomes into approximately 1 Mb semi-independent genetic regions, estimating local genetic correlations using significant regions for each phenotype (0.05/2495) (Werme et al., 2022). Segments with PPA>0.5 were also considered locally associated areas. We converted these segments into cytogenetic location using the R package “biomaRt” (version 2.56.1) (Durinck et al., 2009).

### Genetic overlap analysis

We quantified the genetic overlap between AF and BC using the bivariate MiXeR approach. This method assumes that only a portion of the variation affects the trait (Frei et al., 2019). Following official recommendations, we excluded SNPs from the MHC region in both phenotypes due to LD structure (Frei et al., 2019). The results of the bivariate MiXeR were represented using a Venn diagram.

### Cross-trait GWAS meta-analysis

We utilized the cross-phenotype association analysis (CPASSOC) to identify common risk SNPs between AF and BC (Li and Zhu, 2017). CPASSOC, particularly Shet, offers robust statistical power in the presence of heterogeneity. Therefore, this study chose Shet for analysis.

A threshold of 5e-8 represents the widely accepted genome-wide significance level (Sangurdekar et al., 2019; Li et al., 2022). Therefore, in CPASSOC, a P-value < 5e-8 was considered significant for SNPs associated with both phenotypes. PLINK is a widely used open-source toolset for genome-wide association analysis, known for its high computational efficiency and comprehensive genetic analysis capabilities (Purcell et al., 2007). We referenced the 1000 Genomes Project phase 3 data, using Shet in PLINK to identify the most related independent SNPs within a 1.0 Mb area for both trait (Purcell et al., 2007; Byrska-Bishop et al., 2022). The specific parameters and their significance are as follows: SNPs with a P-value ≤5 × 10^−8^ (–clump-p1) were selected as index SNPs, and SNPs within a ±1 Mb range (–clump-kb 1,000) were searched for with a P-value ≤1 × 10^−5^ (–clump-p2) and LD r^2^ ≥ 0.01 (–clump-r2) with the index SNP. These SNPs were grouped into the index SNP’s clump and excluded. Finally, only independent representative SNPs were retained. SNPs with a CPASSOC P < 5e-8 and a single-trait P < 1e-3 were considered pleiotropic. Additionally, SNPs with a P single-trait > 5e-8 and not previously associated with BC or AF were considered new, unreported pleiotropic SNPs (Wu et al., 2023a).

Lastly, we performed functional annotation of identified pleiotropic SNPs using the Ensembl Variant Effect Predictor (VEP) and 3DSNP (Zerbino et al., 2018; Lu et al., 2017). VEP selects candidate genes based on simple physical proximity (Zerbino et al., 2018). 3DSNP annotates the regulatory functions of SNPs by exploring their 3D interactions with genes mediated by chromatin (Lu et al., 2017).

### Colocalization analysis and fine mapping credible set analysis

To explore whether the same variants cause the two traits, we employed Coloc for colocalization analysis of pleiotropic SNPs (Giambartolomei et al., 2014). This method provides posterior probabilities for five mutually exclusive hypotheses regarding causal variant sharing in a genomic region. We extracted summary statistics for variants within a 500 kb range of each shared pleiotropic SNP and calculated the posterior probability of PPH4 (the probability of both traits being associated through a shared single causal variant). If PPH4 > 0.75, the locus was considered colocalized. In fine-mapping analysis, PLINK and the 1000 Genomes Project reference panel are required for LD estimation. Additionally, we calculated R^2^ values for SNPs within a 500 Kb range of each pleiotropic SNP (Purcell et al., 2007; Byrska-Bishop et al., 2022). Using the Bayesian fine-mapping algorithm FM-summary (https://github.com/hailianghuang/FM-summary), we determined a 99% credible set of causal SNPs for each pleiotropic SNP within this range, aiming to provide reliable targets for downstream experiments (Farh et al., 2015).

### Tissue and cell type enrichment analysis

To identify tissues and cell types closely related to the shared genes, we used the online tool PCGA (https://pmglab.top/pcga) to analyze enrichment in 54 human tissues and cell types from tissues closely associated with AF and BC such as Artery, Blood, Breast, Endothelial cells, Heart, Immune cell, and Immune system (Xue et al., 2022). PCGA employs an iterative estimation framework based on the method of driver tissue estimation by selective expression. It has collected data from 54 types of human tissues via gtxv8 and single-cell RNA data from 2,214 cell types. By integrating GWAS summary statistics and transcriptome data, it effectively estimates related tissue/cell types and genes. Cell enrichment P-values were corrected using False Discovery Rate Correction (FDR).

### Transcriptome-wide association studies (TWAS)

Considering that genetic variations can influence traits by affecting gene expression, we conducted TWAS to identify overlapping genes potentially having causal relationships. We used Joint-tissue imputation (JTI) models based on multi-tissue transcriptomic data from GTEx v8 comprising 49 tissues (Zhou et al., 2020; Consortium, 2013). This analysis utilized pre-trained expression quantitative trait locus (eQTL) models and GWAS summary statistics. This method accounts for shared genetic effects across different tissues and unique genetic regulation in target tissues. Compared to models like PrediXcan and UTMOST, JTI significantly enhances predictive capability (Zhou et al., 2020). We conducted TWAS using the JTI models for 49 tissues, adjusting p-values within each tissue using FDR.

### TWAS fine mapping

We employed Fine-mapping of causal gene sets (FOCUS) to further evaluate the significance of genes identified in the TWAS (Mancuso et al., 2019). FOCUS utilizes predicted eQTL weights, LD, and GWAS summary data to estimate potential pathogenic genes from TWAS. It uses a Bayesian algorithm to assess the posterior inclusion probability (PIP) of each feature in the association region. We utilized GTEx v8 eQTL weights from FUSION across 49 tissues (Gusev et al., 2016). Genes with significant p-values in the JTI results and a PIP >0.5 in FOCUS analysis were considered potential pathogenic genes.

### Phenome-wide association studies (phewas)

We conducted a Phewas of the newly identified pleiotropic SNPs using an online tool (https://gwas.mrcieu.ac.uk/phewas/) with a threshold of P < 5e-8 (Elsworth et al., 2020). Additionally, we performed a Phewas of the genes identified by TWAS using publicly available data from the GWAS Atlas (https://atlas.ctglab.nl) for 4756 phenotypes with a significance threshold of P < 0.05/4756.

### Protein-protein interaction (PPI)

To determine if there are interrelationships among the identified pleiotropic genes, we analyzed relevant genes annotated by VEP and 3DSNP, and pleiotropic genes identified through TWAS using STING (https://string-db.org/) (von Mering et al., 2003).

### Causal inference

Genetic correlation may be due to pleiotropy or causal relationships (Zhu et al., 2015). Pleiotropy represents the same genetic variation affecting two traits simultaneously. In contrast, causal relationships imply that genetic variation can influence one trait and thereby affect another.

To explore the genetic causal relationship between AF and BC, we designed a Mendelian randomization (MR) analysis based on the STROBE-MR guidelines (Supplementary Materials). We used software packages such as “TwoSampleMR,” “RadialMR,” “CAUSE,” “GSMR,” and “MendelianRandomization” to perform MR analysis on GWAS data. MR analysis relies on three core assumptions (Zheng et al., 2017): (1) the instrumental variables (IVs) is closely associated with the exposure; (2) the IVs is unrelated to confounders; (3) the IVs affects the outcome only through the exposure (Figure 1F).

Initially, we performed univariate MR analysis using seven methods at a threshold of P < 5e-8, including inverse variance weighting (IVW), weighted median, MR-Egger, weighted mode, simple mode, GSMR, CAUSE. Before MR analysis, LD was removed using 1,000 Genomes reference data. Subsequently, we used the GWAS catalog to eliminate SNPs related to known common confounders such as obesity, smoking, and alcohol consumption (Sollis et al., 2023). Sensitivity analyses were then conducted to remove outliers to stabilize results. MR-Egger intercept testing and Cochran’s Q statistic assessed pleiotropy and heterogeneity. Outliers were removed using MR-Presso, radial IVW, and radial Egger. In univariate MR analysis, IVW served as the primary outcome. GSMR analysis considered potential LD among SNPs (Zhu et al., 2018). CAUSE controls for potential pleiotropy, significantly reducing the false-positive rate (Morrison et al., 2020). We included these methods to aid in interpreting results. Finally, a leave-one-out analysis was performed to detect influential SNPs, and statistical power was calculated using mRnd (Brion et al., 2013).

To better manage potential confounders, we performed an additional multivariable MR (MVMR) analysis. This analysis incorporated phenotypes related to obesity from the FinnGen research project (R10) (Kurki et al., 2023). It also included phenotypes for smoking initiation and alcohol consumption from the GWAS and Sequencing Consortium of Alcohol and Nicotine use [GSCAN] study, excluding samples from the United Kingdom Biobank (Liu et al., 2019). MVMR-IVW was the primary outcome, capable of detecting potential outliers and pleiotropy. Additional methods like MVMR-Lasso, MVMR-Egger, and MVMR-Median were used to consolidate results.

## Results

### Global genetic analysis

In the unrestricted LDSC analysis, the results indicated a potential genetic correlation between AF and BC with rg = 0.0435 and P = 0.0388 (Figure 1B). Although the HDL analysis did not reach a significant threshold, it also showed a positive correlation between AF and BC (rg = 0.0319, P = 0.101, Table 1).

**TABLE 1:** Results of the global genetic analysis between AF and BC.

	Atrial fibrillation	Breast cancer	P value
Heritability (standard error)			
LDSC	0.0259 (0.0033)	0.1295 (0.0117)	
LDSC-no intercept	0.0286 (0.0035)	0.1618 (0.0123)	
HDL	0.0227 (0.0037)	0.149 (0.0126)	
Genetic Correlation (standard error)			
LDSC	0.0435 (0.021)		0.039
LDSC-no intercept	0.0293 (0.0147)		0.047
HDL	0.0319 (0.0194)		0.101

LDSC, linkage disequilibrium Score; HDL, high-definition likelihood

### Local genetic analysis

**FIGURE 2:**
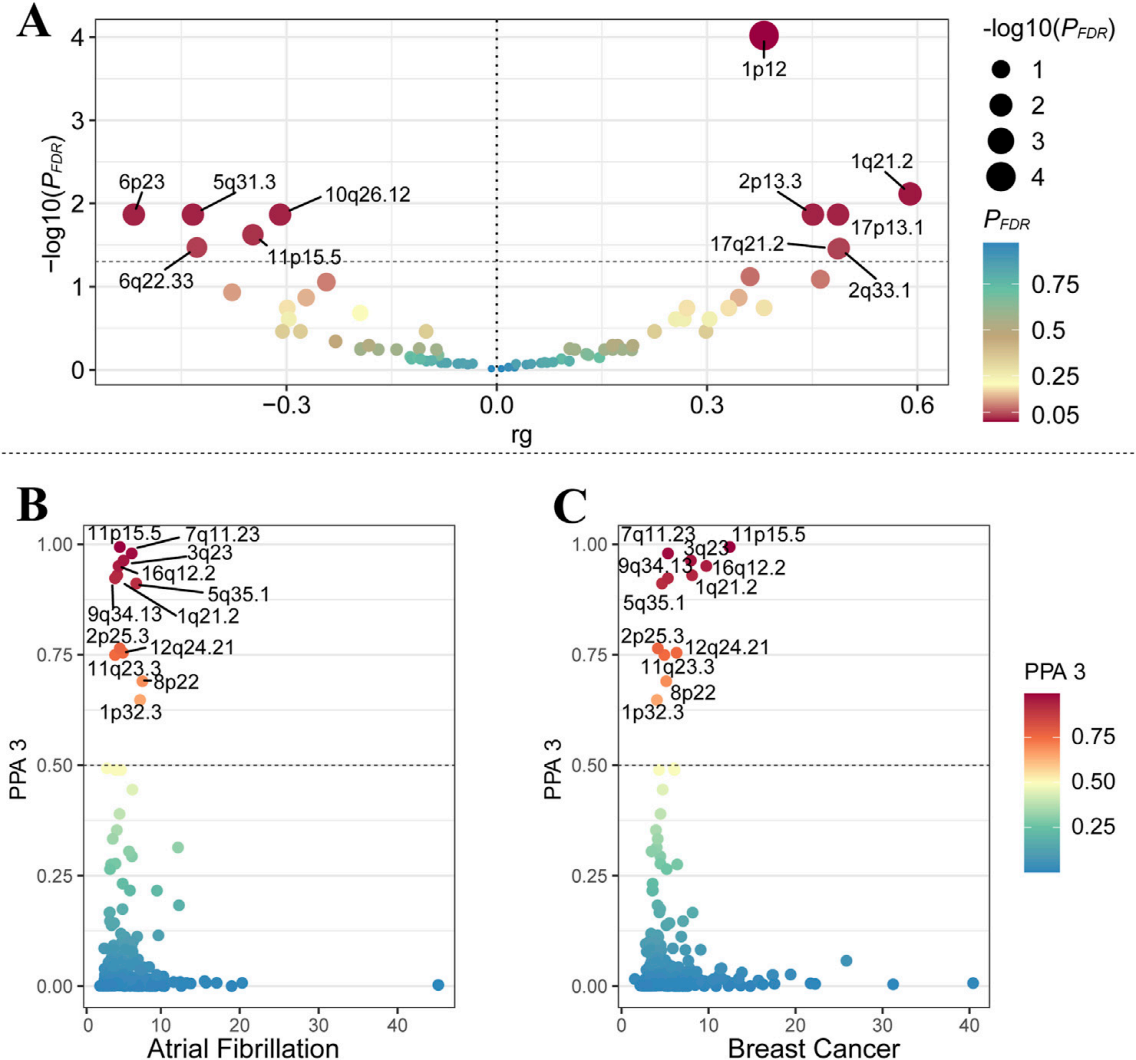
**(A)** represents the results of the LAVA analysis, with the X-axis representing genetic correlation and the Y-axis representing the -log10 (*P*_*FDR*_) values. The dashed line represents -log10 (0.05). **(B, C)** respectively represent the results for AF and BC from the GWAS-PW analysis, with the X-axis showing the maximum absolute value of the Z-score, and the Y-axis displaying data for PPA 3.

In reference to the results of global genetic analysis, we analyzed the local genetic characteristics between AF and BC. After FDR correction, LAVA identified 11 significant regions (P_FDR_ < 0.05, Supplementary Table S2). GWAS-PW identified 12 significant regions with PPA3 > 0.05 between AF and BC (Supplementary Table S3). Notably, the region near 11p15.5 (chromosome 11: 1,857,846-2,477,449) was consistently validated by both methods (Figure 2).

### Genetic overlap

**FIGURE 3:**
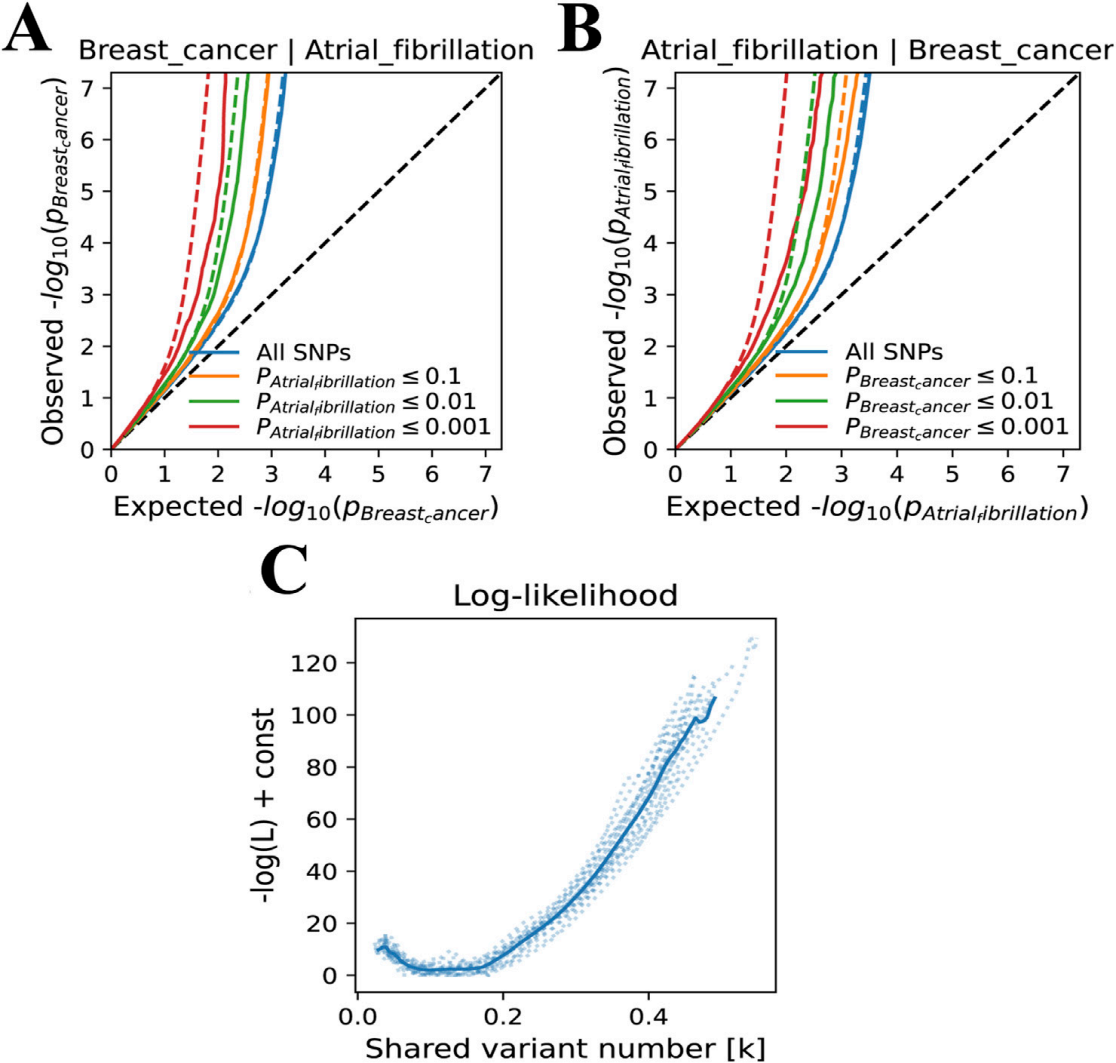
**(A)** A Venn diagram represents the data on the potential pleiotropic genetic overlap between AF and BC from the MiXer results. **(B)** QQ plots represent the results stratified by P-values for BC and AF. **(C)** The negative log-likelihood plot shows a minimum model score of about 15, a maximum score of about 110, and the best model score close to 0, indicating a good fit of the model.

In the bivariate MiXer analysis excluding the MHC region, the rg between the two was 0.05. MiXer predicted 348 variants affecting AF and approximately 568 for BC, with about 125 shared variants between them (Figure 1C). The bivariate stratified QQ plot for AF and BC showed a significant left shift in the groups of SNPs with higher significance (Figure 3), strengthening the notion of shared genetic overlap between AF and BC.

### Cross-trait GWAS meta-analysis, pleiotropic loci, and colocalization analysis

Given the observed genetic overlap between AF and BC, we used CPASSOC to explore individual genetic variations. At a genome-wide threshold (P < 5e-8), CPASSOC identified 323 independent SNPs (Supplementary Table S4), including 14 pleiotropic SNPs and 1 new pleiotropic SNP (Table 2). Of the total 15 pleiotropic SNPs, 8 SNPs representing loci were supported by colocalization (PP.H4 > 0.75, N = 8; PP.H4 > 0.9, N = 7), suggesting these loci as key in influencing both traits, reinforcing the causal association between the loci and traits (Supplementary Figures S1–S8). This includes a new pleiotropic SNP, rs114414434, which colocalization results also suggest as a key site affecting both traits (PP.H4 = 0.794, Table 2). At a genome-wide threshold (P < 5e-8), the Phewas results for the newly identified pleiotropic SNP rs114414434 show significant associations with high expression of ENSG00000152684 (P = 2.76e-15) and ENSG00000213949 (P = 2.20e-08) (Supplementary Table S5).

**TABLE 2:** Annotation and colocalization analysis results of 15 pleiotropic SNPs.

SNP	Band	Symbol	Z value		P value			PP.H4	3D.interacting.gene
			BC	AF	BC	AF	Shet		
rs146518726	1p32.3		3.4	7.8	0.000725	8.27E-15	7.09E-16	0.181	
rs11205303	1q21.2	MTMR11	8.1	4.1	5.07E-16	3.38E-05	3.20E-15	0.931	HIST2H2AB and other 8
rs7578393	2p23.3	KIF3C	3.8	7.0	0.000137	2.42E-12	1.32E-13	0.519	KIF3C and other 4
rs6440006	3q23	ZBTB38	8.0	5.0	1.59E-15	7.07E-07	9.85E-15	0.972	
rs114414434	5q11.2		3.7	−5.0	0.00019	7.26E-07	4.15E-08	0.794	
rs56180201	5q35.2	CPEB4	−3.9	−6.9	8.06E-05	8.09E-12	2.13E-13	0.944	C5orf47 and other 2
rs35005436	7q11.23	GTF2I	5.1	6.3	4.11E-07	3.34E-10	2.07E-12	0.982	
rs56201652	7q21.2	CDK6	3.5	−7.1	0.00047	1.74E-12	6.15E-14	9.94E-05	FAM133B and other 2
rs12245149	10q21.3	REEP3	4.1	−7.0	4.01E-05	1.66E-12	3.18E-14	0.036	JMJD1C and other 2
rs2936870	10q26.13	FGFR2	−39.2	4.1	<5e-324	3.75E-05	<5e-324	0.932	FGFR2
rs1973765	11p15.5	LSP1	−12.4	4.3	1.77E-35	1.63E-05	2.19E-34	0.963	SYT8 and other 12
rs1061657	12q24.21	TBX3	6.3	4.4	2.52E-10	1.12E-05	1.22E-09	1.89E-06	TBX3
rs6598541	15q26.3	IGF1R	−3.4	−6.3	0.000678	2.22E-10	2.21E-11	0.338	IGF1R,MIR4714
rs62048402	16q12.2	FTO	−9.7	4.4	1.94E-22	1.38E-05	1.57E-21	0.924	RPGRIP1L and other 3
rs2696608	17q21.31	KANSL1	−6.1	6.1	1.24E-09	1.12E-09	7.65E-13	0.394	

SNP, single nucleotide polymorphism; Band, Cytogenetic location; AF, atrial fibrillation; BC, breast cancer; Symbol, The gene physically closest to the SNP, annotated by VEP; 3D.interacting.gene, The gene interacting with the variant, annotated by 3DSNP.

For each pleiotropic SNP, we identified a 99% credible set of causal SNPs within a 500 kb range, totaling 2,035 SNPs related to AF and BC (Supplementary Table S6).

### Tissue and cell type enrichment

**FIGURE 4:**
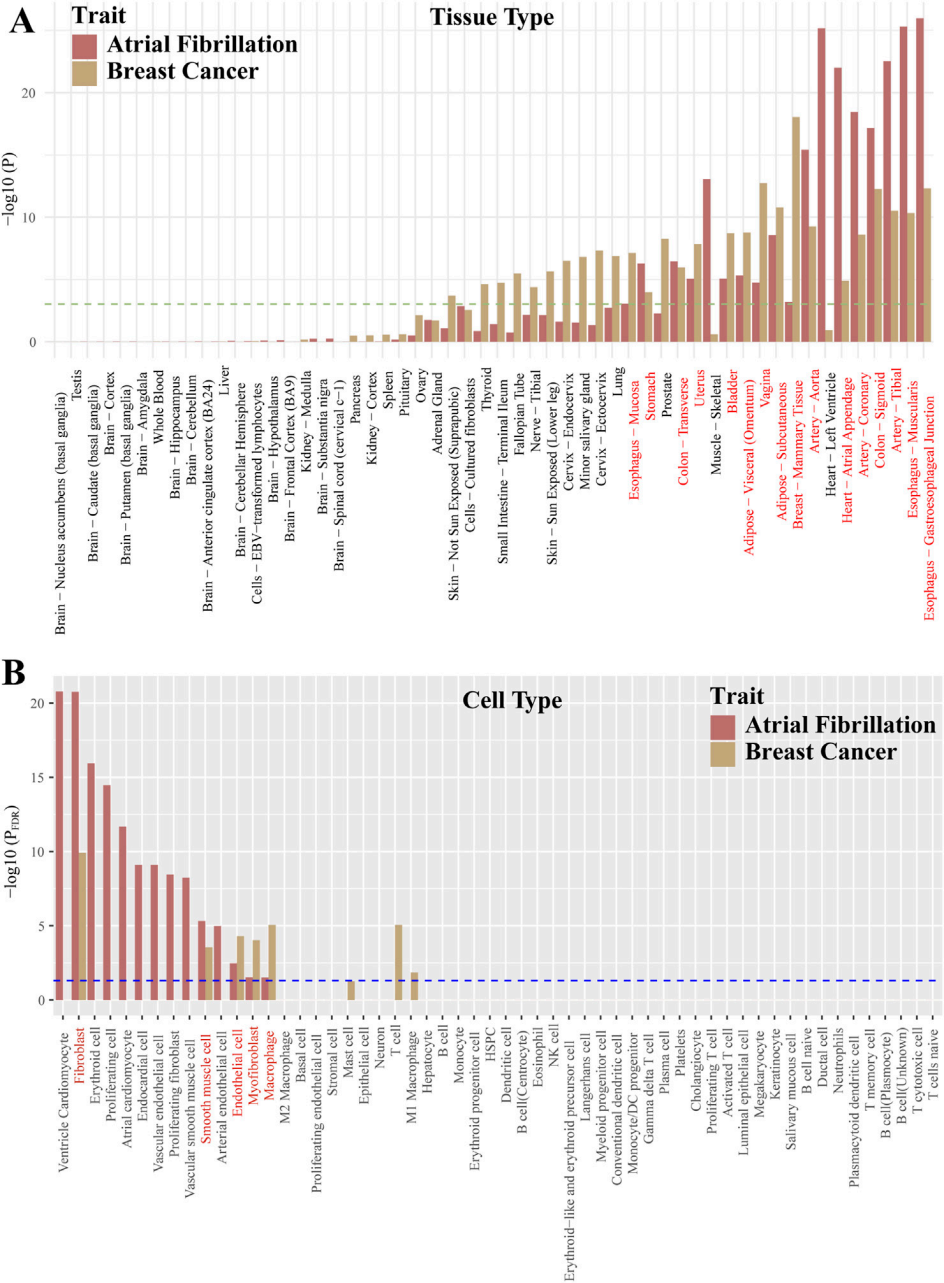
Co-enriched tissues and cell types between atrial fibrillation and breast cancer. Red font indicates co-enrichment. **(A)**: Represents the tissue types. **(B)**: Represents the cell types.

Our findings indicate that AF and BC are jointly enriched in 16 tissues including “Breast - Mammary Tissue,” “Vagina,” “Artery - Tibial,” “Artery - Aorta,” “Artery - Coronary,” “Uterus,” “Heart - Atrial Appendage,” “Colon” (Figure 4A; Supplementary Tables S7–S9). Moreover, cell type enrichment analysis in disease-related tissues such as Artery, Blood, Breast, Endothelial cells, Heart, Immune cell, Immune system showed joint enrichment in Fibroblast, Smooth muscle cell, Endothelial cell, Myofibroblast, Macrophage (Figure 4B; Supplementary Tables S10, S11).

### TWAS analysis and TWAS fine mapping

Using JTI models across 49 tissues, we identified 1,276 genes (P_FDR_ < 0.05), reducing to 205 unique genes after accounting for tissue origin (Supplementary Table S12). The FOCUS method identified 61 genes across 49 tissues (PIP >0.5), reducing to 30 unique genes after considering tissue origin (Supplementary Table S13). Of these, 23 genes confirmed by both methods, were reduced to 10 unique genes after removing duplicates (Supplementary Table S14). These included “GMCL1,” “DNMT3A,” “SNRNP27,” “NRBF2,” “FBXO32,” “JMJD1C,” “GTF2IRD2,” “ANXA4,” “RUSC1-AS1,” “GTF2I.”

Phewas associated nine genes (ANXA4, DNMT3A, FBXO32, GMCL1, GTF2I, GTF2IRD2, JMJD1C, NRBF2, SNRNP27) with 459 phenotypes under a threshold of P < 0.05/4756, with the most frequently associated traits being body mass index (N = 23), Height (N = 9), and educational attainment (N = 8) (Supplementary Table S15).

Among the total of 30 relevant genes identified by VEP, 3DSNP annotations, and TWAS, PPI analysis at a confidence score threshold of 0.15 showed that DNMT3A had the most associations with other genes (Figure 1D).

### Causal relationships

**FIGURE 5:**
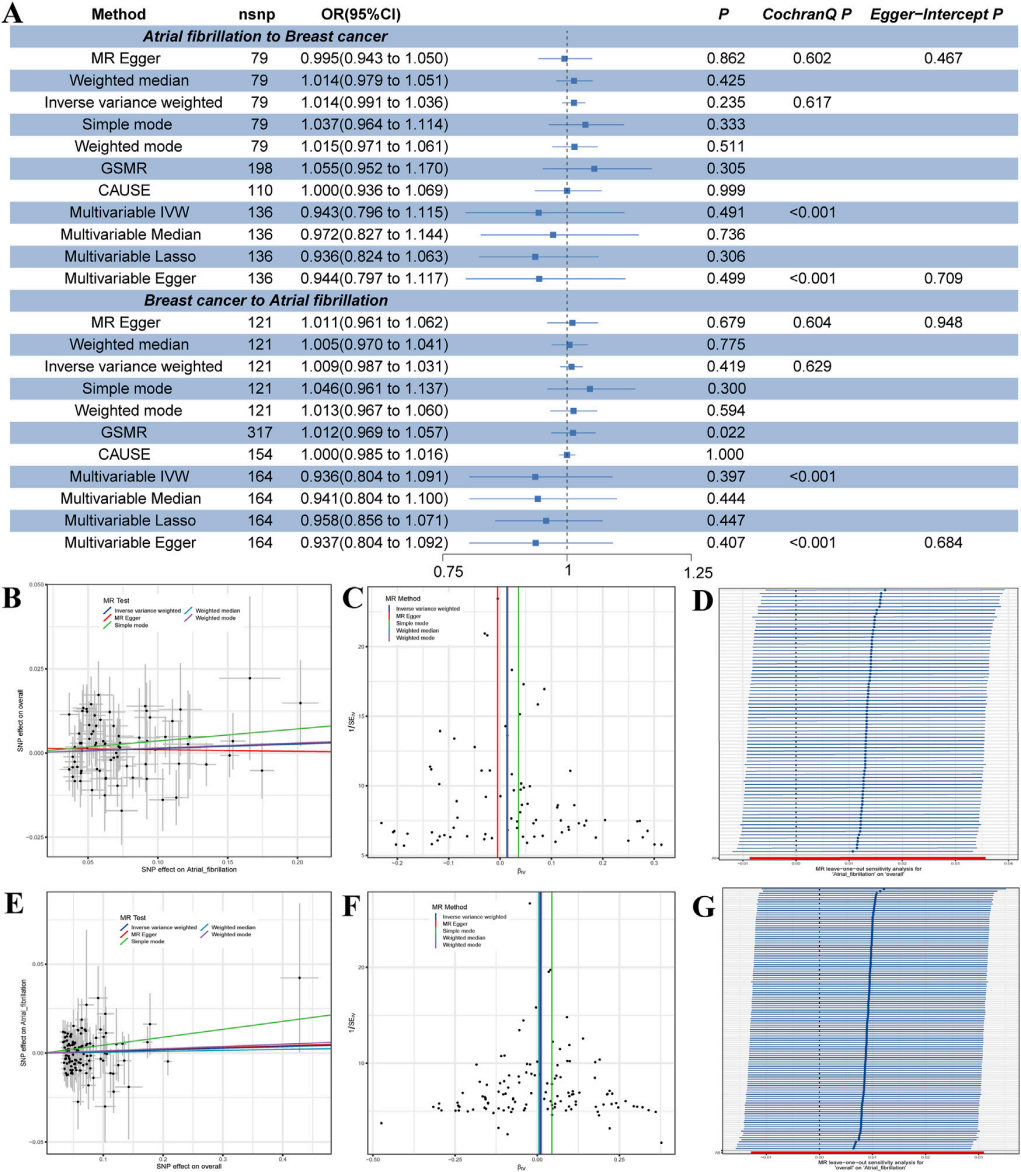
**(A)** Results of univariate and multivariate Mendelian randomization analyses between AF and BC. This includes 7 methods of univariate MR analysis and 4 methods of multivariate MR analysis. **(B)** Scatter plot of univariate MR analysis from AF to BC. **(C)** Funnel plot of univariate MR analysis from AF to BC. **(D)** Leave-one-out (LOO) plot of univariate MR analysis from AF to BC. **(E)** Scatter plot of univariate MR analysis from BC to AF. **(F)** Funnel plot of univariate MR analysis from BC to AF. **(G)** LOO plot of univariate MR analysis from BC to AF.

Finally, we predicted the causal relationship between AF and BC from a genetic perspective using two-sample Mendelian randomization. We used 79 A F-related SNPs as IVs to test for potential causal effects of AF on BC (F-statistic from 29.7–510.6, Supplementary Table S16) and 121 BC-related SNPs as IVs for potential causal effects of BC on AF (F-statistic from 29.8-958.4, Supplementary Table S17). The F-statistic >10, avoiding bias from weak IVs (Burgess et al., 2011). Univariate MR results indicated no positive or negative causal relationship between AF and BC (Figure 5A). Results from CAUSE, GSMR, and LOO analyses reinforced this view (Supplementary Figures S10–S15; Supplementary Tables S18, S19). Statistical power is detailed in Supplementary Table S20.

In MVMR analysis incorporating major confounders such as obesity, alcohol use, and smoking, we used random effects IVW, which allows for heterogeneity in sensitivity tests (Papadimitriou et al., 2020). By incorporating SNPs associated with relevant confounding factors into the MVMR, the final MVMR results, using 136 SNPs as IVs for AF and 164 SNPs for BC, still showed no positive or negative causal relationship between AF and BC (Figure 5).

## Discussion

In previous epidemiological studies, while a link between AF and BC was observed, the mechanisms underlying this association were unclear. Therefore, we utilized the largest GWAS summary data available for AF and BC to systematically investigate their potential shared genetic structures from both SNP and gene levels. To our knowledge, this is the first comprehensive study to examine the shared genetic architecture between AF and BC. Our results demonstrate significant shared genetics between AF and BC in both global and local genetic analyses. We further analyzed the pleiotropy and causality of these genetic overlaps, ultimately identifying multiple pleiotropic SNPs and genes. Moreover, our findings indicate no causal relationship between AF and BC from a genetic standpoint, which partially explains the epidemiological link observed between these conditions. Our approach from the SNP and gene perspective offers new insights into the association and causality between these conditions.

Previous meta-analysis has elucidated the bidirectional association between AF and BC (Yao et al., 2023). Potential mechanisms include systemic inflammation, with elevated inflammatory markers (such as CRP, TNF-α, and IL-6) in BC patients, and AF sharing pathways with the NLRP-3 inflammasome (Yao et al., 2018; Scott et al., 2019; Ershaid et al., 2019). Furthermore, cancer treatments, such as anthracyclines and trastuzumab, can lead to AF (Leong et al., 2016; Khouri et al., 2012). Conversely, AF medications, such as amiodarone and digoxin, may increase the risk of BC (Fares, 2013; Biggar et al., 2011). However, there is currently a lack of relevant studies from a genetic perspective. Therefore, this study is the first to explore the association between AF and BC from a genetic perspective, providing new insights into the shared pathogenesis of these two diseases, which has important implications for future clinical prevention and treatment strategies.

Our initial analysis using unconstrained intercept LDSC identified a significant overall genetic correlation between AF and BC. To solidify these results and considering no sample overlap, we employed constrained intercept LDSC and HDL methods. Constrained intercept LDSC also indicated significant genetic correlation, and HDL suggested a positive genetic correlation between AF and BC. These findings were corroborated by MiXer (rg = 0.05), which revealed potential genetic overlap outside the MHC region. In local genetic correlation analysis, both LAVA and GWAS-PW highlighted significant regions, notably around 11p15.5 (chr11: 1,857,846-2,477,449). Using the R package “biomaRt,” we identified 27 genes within this segment, including MIR483, LSP1, IGF2, which are associated with the onset and progression of BC (Luo et al., 2021; Hemminki et al., 2010; Cui et al., 2019), and H19, KCNQ1, linked to AF (Chen et al., 2003; Guo et al., 2022).

Considering the pleiotropic genetic variations and genes that can simultaneously affect AF and BC, we initially identified 15 pleiotropic SNPs at the SNP level. Among these 15 pleiotropic SNPs, rs2936870 located near 10q26.13 represented the strongest shared signal (PShet < 5e-324, PP.H4 = 0.932). Multiple studies have reported its close association with BC (Lei and Deng, 2017; Xu et al., 2011). Research has also found that in mouse models, it plays an important role in the recovery of cardiac function and vascular remodeling after myocardial ischemia-reperfusion injury (House et al., 2016). Among other pleiotropic SNPs, rs146518726, rs11205303, rs6440006, rs1973765, rs2936870, and rs35005436 are all located at loci with positive genetic correlations. In the Phewas of the new pleiotropic SNPs, they are shown to be related to the expression of two genes. ENSG00000152684 (PELO) is involved in regulating the ATPase activity of the NOD-like receptor family, controlling its oligomerization assembly and activation, thus participating in the regulation of various immune inflammatory responses mediated by the NLR family (Wu et al., 2023b). Studies related to ENSG00000213949 (ITGA1) have found that elevated levels of ITGA1 are closely associated with cardiac dysfunction in type 2 diabetes (Su et al., 2024) and also linked to the development of various cancers (Boudjadi et al., 2016). However, more detailed functional studies are needed for these pleiotropic sites to help us discover their relationship with AF and BC.

Then, we explored possible pleiotropic genes at the gene level using JTI and FOCUS methods. Among the 10 pleiotropic genes identified by TWAS, ANXA4 is reported to be a key immune-related gene in AF (Yan et al., 2021). Moreover, ANXA4 can activate the JAK-STAT3 signaling pathway by increasing the expression of JAK1 and phosphorylation of STAT3, ultimately affecting the progression of BC (Li et al., 2020). FBXO32 has been reported to be associated with BC risk (Wang et al., 2021). In diabetic mouse models, FBXO32 was found to regulate the expression of small conductance calcium-activated potassium channel 2 (SK2) in the atria, thereby increasing the risk of AF (Ling et al., 2019). Studies have found that silencing of the NRBF2 promoter may be related to the development of breast cancer (Darabi et al., 2015). Additionally, variations within the NRBF2 region have been found to have a significant statistical correlation with the risk of AF in European and East Asian populations (Hong et al., 2021). Changes in the expression of the SNRNP27 gene may play a role in the susceptibility to AF (Hsu et al., 2018). SNRNP27, as one of the potential targets of miR-146b-5p, may play a role in liver inflammation and liver cancer (Kirchmeyer et al., 2018). Mutations in DNMT3A are more common in patients with AF than in individuals without it (Ahn et al., 2024). DNMT3A may promote cardiac fibrosis by silencing RASSF1A, thereby activating the ERK1/2 signaling pathway, which may lead to AF (Tao et al., 2014). In young women receiving BC treatment, DNMT3A is one of the most common mutated genes (Gibson et al., 2023). The PPI results also indicate that DNMT3A could be a key gene between BC and AF. In other genes, such as GTF2I, JMJD1C, RUSC1-AS1, while reported to be associated with the development of BC, they have not been reported in relation to AF (Watanabe et al., 2013; Li et al., 2019; Niinaka et al., 2010). Furthermore, we identified two potential new pleiotropic genes, including GMCL1 and GTF2IRD2. GTF2IRD2 is thought to be derived from the GTF2I sequence (Makeyev et al., 2004), and PPI results reinforce this view. GTF2IRD2 has been reported to play a crucial role in the pathogenesis of Williams-Beuren syndrome (Makeyev et al., 2004). GMCL1 is considered a candidate tumor suppressor gene, and its encoded protein is involved in the control of the MDM2-P53 axis (Masuhara et al., 2003). Abnormal expression of the GMCL1 gene has been found to affect the prognosis of diffuse large B-cell lymphoma (Fournier et al., 2010). In the Phewas of these pleiotropic genes, we found that these genes were most strongly associated with BMI, height, and educational attainment. Furthermore, obesity and height are currently considered independent risk factors for AF and BC (Koene et al., 2016; Aune et al., 2017; Kofler et al., 2017; Gremke et al., 2022), suggesting that the epidemiological observation of a link between AF and BC may be due to risk factors such as obesity. Furthermore, TWAS results and tissue and cell enrichment results show that the shared pathways between AF and BC may extend to multiple systems such as the digestive, reproductive, and circulatory systems. For example, it has been found that gut microbiota can increase the risk of AF or BC (Li et al., 2023; Zhang et al., 2021). In addition, among the 5 cell types enriched in common between AF and BC, macrophages have been reported to participate in tissue regeneration and myocardial injury (Leanca et al., 2022). Resident cardiac macrophages are a subtype of self-renewing cells that can reproduce to protect the myocardium (Sansonetti et al., 2020). In breast development, there is also a resident macrophage subtype (Boutas et al., 2023). Resident macrophages have been found to facilitate the metastatic cascade and growth of tumors (Nalio Ramos et al., 2022). These study results indicate that the relationship between AF and BC may be very complex. The diseases share many common risk genes, which can genetically explain the link between AF and BC. According to the enrichment results, the link between the two can even be traced back to the origins of related tissues and cell types. However, more research is needed to discover the pathophysiological mechanisms between the two.

In the use of genetic tools to predict causal relationships, there is no potential causal link between AF and BC. This result suggests that the potential link between AF and BC may be caused by common risk factors, related treatments for AF or BC, or common pleiotropic genetic outcomes, such as the use of amiodarone, which has been found to be related to an increased risk of cancer (Wassertheil-Smoller et al., 2017; Su et al., 2013). A recent study has shown that young BC patients undergoing breast-conserving surgery have a higher risk of AF, and the use of anthracycline drugs may also be associated with an increased risk of AF (Park et al., 2024). It is noteworthy that these results may mask the potential inherent association between AF and BC, causing us to overlook the inherent link between AF and BC. Our study results indicate that there is some genetic overlap shared between AF and BC. Our study utilizes the largest available AF and BC GWAS summary data to systematically investigate their concurrent relationship, revealing shared genetic pleiotropy rather than a direct causal link. By identifying pleiotropic genes and pathways, our findings suggest potential biological mechanisms that may contribute to the observed epidemiological association. These results provide valuable targets for future research, guiding efforts toward understanding the molecular basis of AF and BC co-occurrence and informing potential risk stratification or therapeutic interventions. Further studies incorporating functional validation and multi-ancestry analyses are warranted to refine these findings and explore their clinical implications.

We acknowledge some limitations in this study. First, our study only uses data from European populations, which does not have universal applicability to other races. Secondly, because the BC population includes only female samples, and due to the limited availability of summary data, we were unable to conduct gender-stratified analysis. Therefore, the distribution of information on the X chromosome is not balanced between the two populations, and most tools used in this study do not support analysis of the X chromosome. Thus, our study did not conduct a more in-depth study of the X chromosome. Third, as this study focuses on the relationship between overall BC and AF throughout the day, it did not further explore the associations between BC subtypes and AF. Finally, more in-depth experimental work and more complete cohort studies are needed to help us better understand the relationship between AF and BC and the pathophysiological mechanisms between them.

## Conclusion

Our study provides a genetic correlation analysis between AF and BC, revealing genetic overlaps and identifying 15 pleiotropic SNPs and 10 pleiotropic genes. We further analyzed cross-enriched tissues and cell types and provided evidence from a genetic perspective using MR and MVMR for a non-causal relationship between AF and BC. Our findings enhance understanding of the potential biological mechanisms between AF and BC, which could influence future research aimed at reducing the risks of both conditions.

## Data Availability

The original contributions presented in the study are included in the article/Supplementary Material, further inquiries can be directed to the corresponding authors.
