# Supplementary material for: Genetic analysis reveals the shared genetic architecture between breast cancer and atrial fibrillation
Source: Front Genet. 2025 Mar 25;16:1450259. doi: 10.3389/fgene.2025.1450259 (PMC11975938; doi:10.3389/fgene.2025.1450259)
Supplement: Supplementary file 3 [file DataSheet1.PDF]

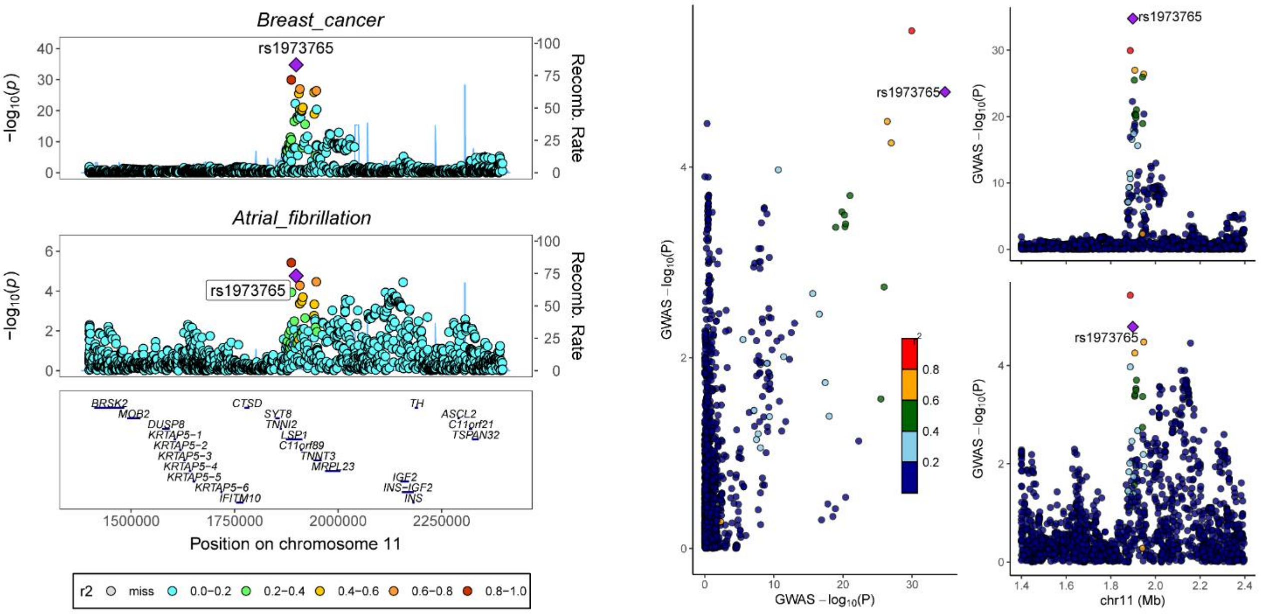

Supplementary Figure 1: Visualization of the colocalization results for the pleiotropic SNP rs1973765.

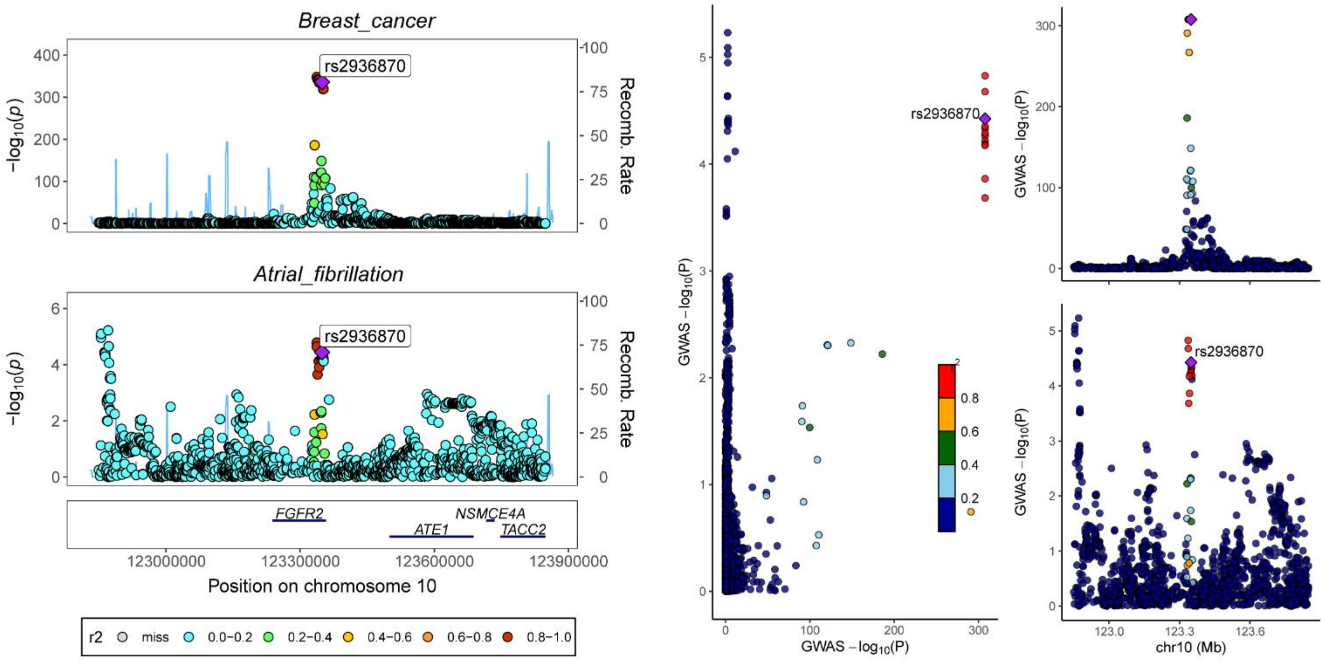

Supplementary Figure 2: Visualization of the colocalization results for the pleiotropic SNP rs2936870.

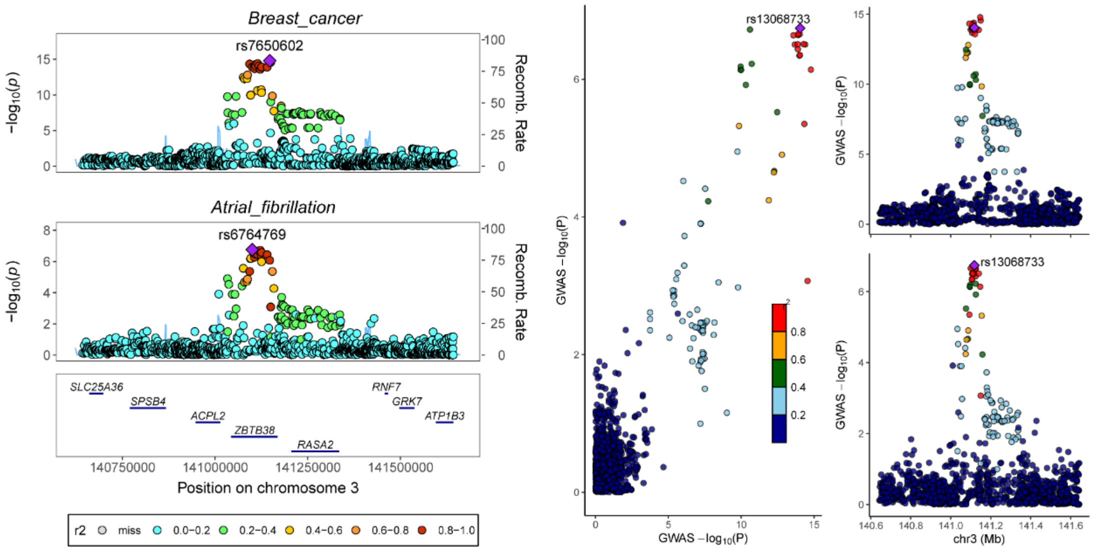

Supplementary Figure 3: Visualization of the colocalization results for the pleiotropic SNP rs6440006.

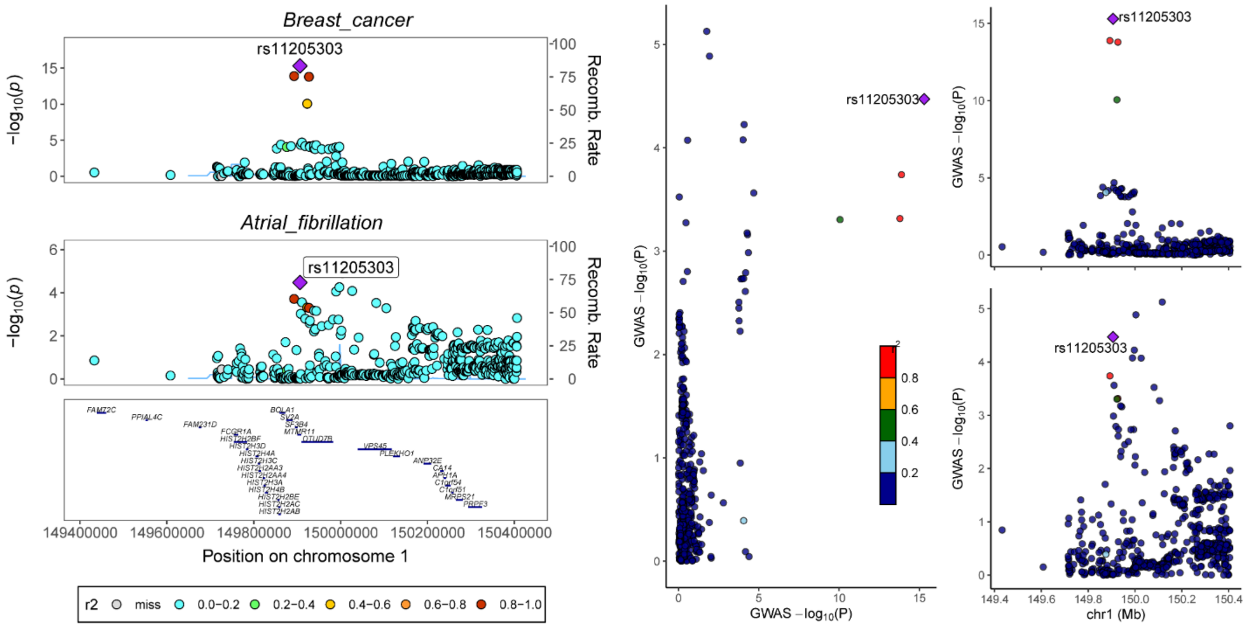

Supplementary Figure 4: Visualization of the colocalization results for the pleiotropic SNP rs11205303.

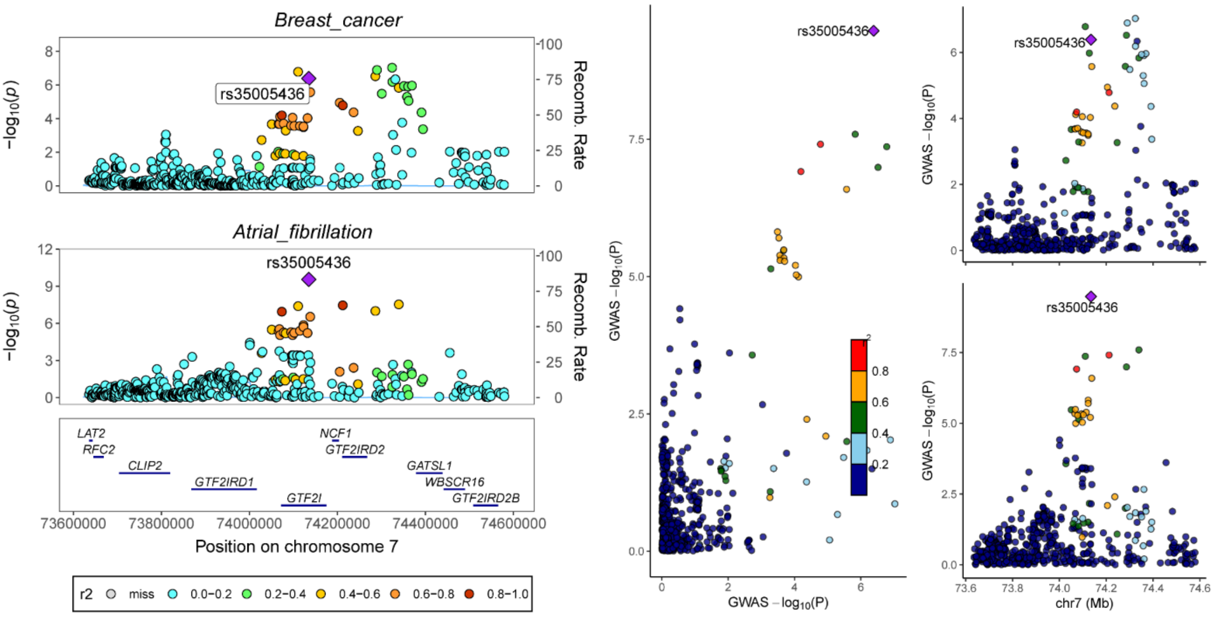

Supplementary Figure 5: Visualization of the colocalization results for the pleiotropic SNP rs35005436.

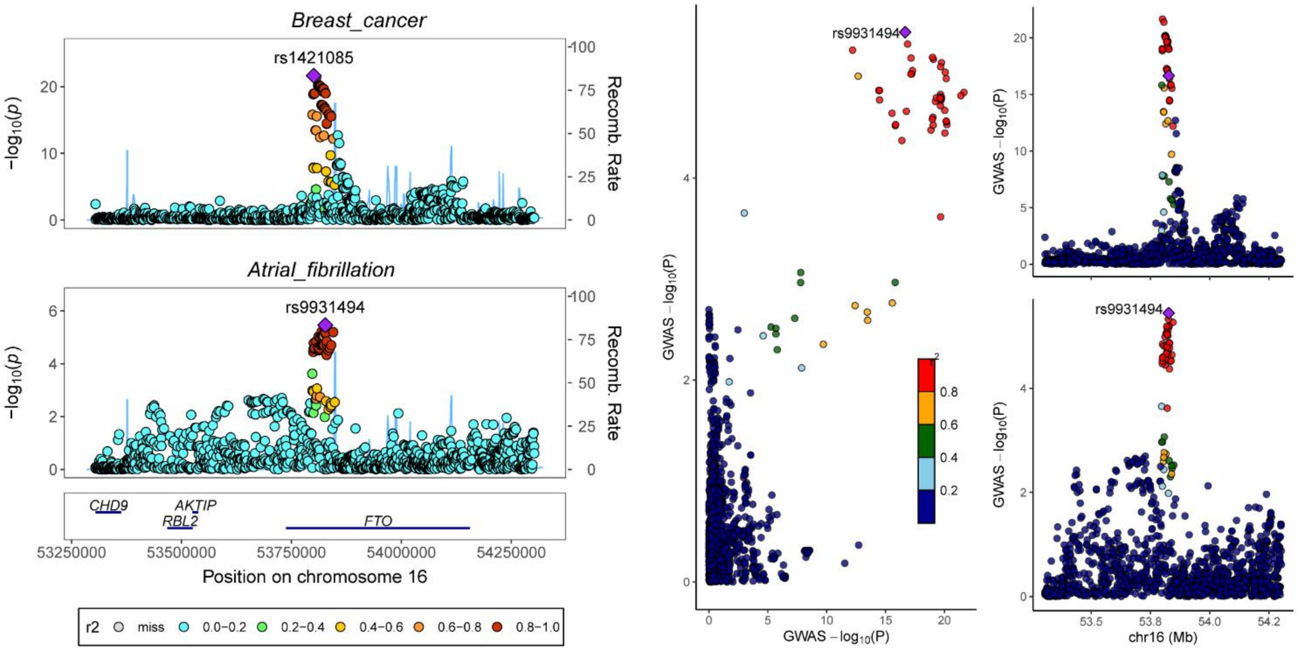

Supplementary Figure 6: Visualization of the colocalization results for the pleiotropic SNP rs62048402.

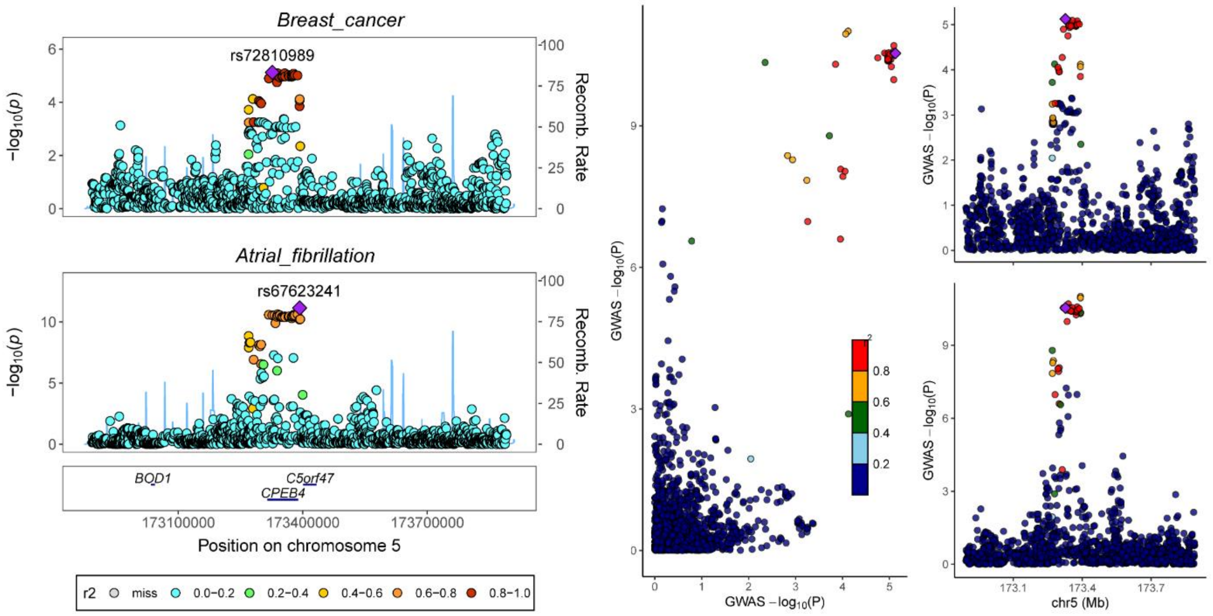

Supplementary Figure 7: Visualization of the colocalization results for the pleiotropic SNP rs56180201.

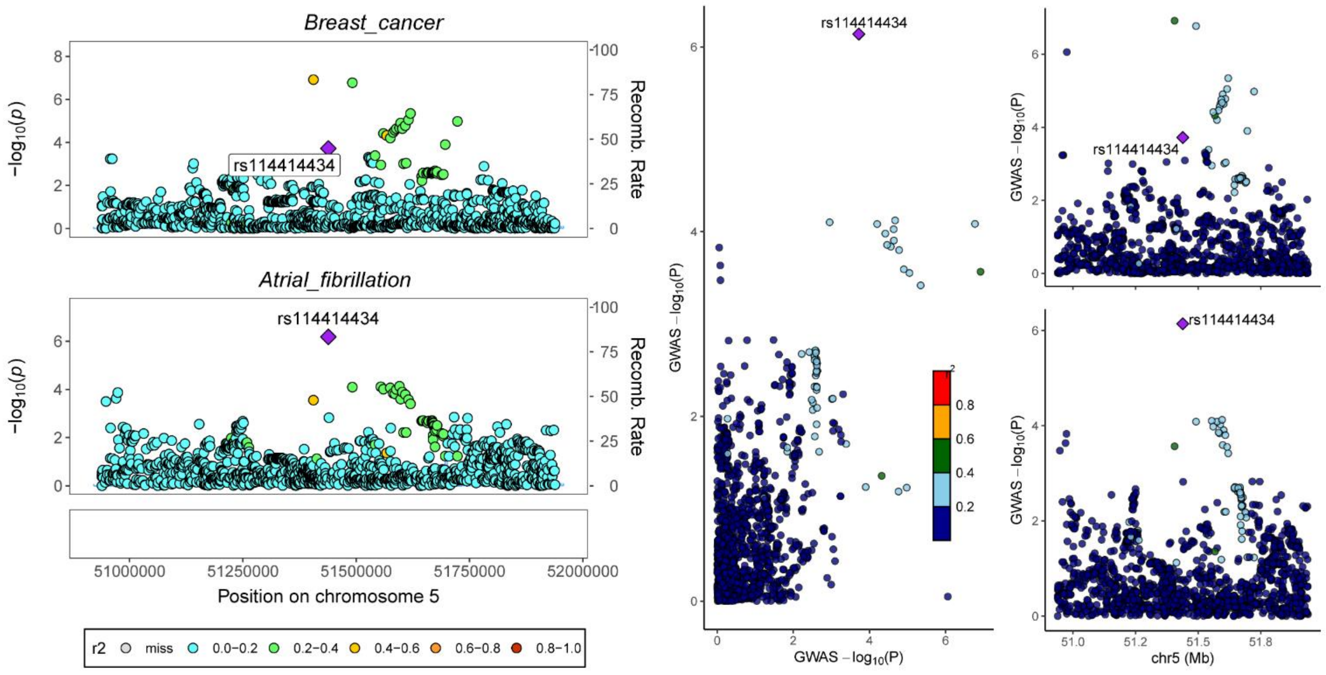

Supplementary Figure 8: Visualization of the colocalization results for the pleiotropic SNP rs114414434.

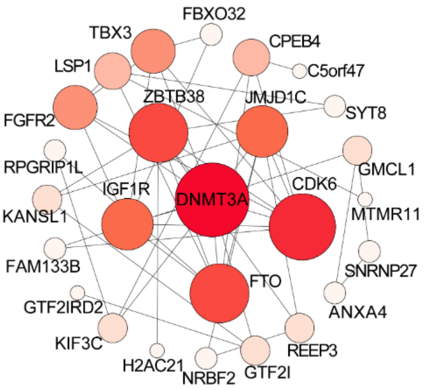

Supplementary Figure 9: PPI network diagram of 30 genes with potential pleiotropy, including 26 protein-coding genes. The size of the circles is proportional to the number of interactions between proteins. The lines between the circles represent the interactions between proteins.

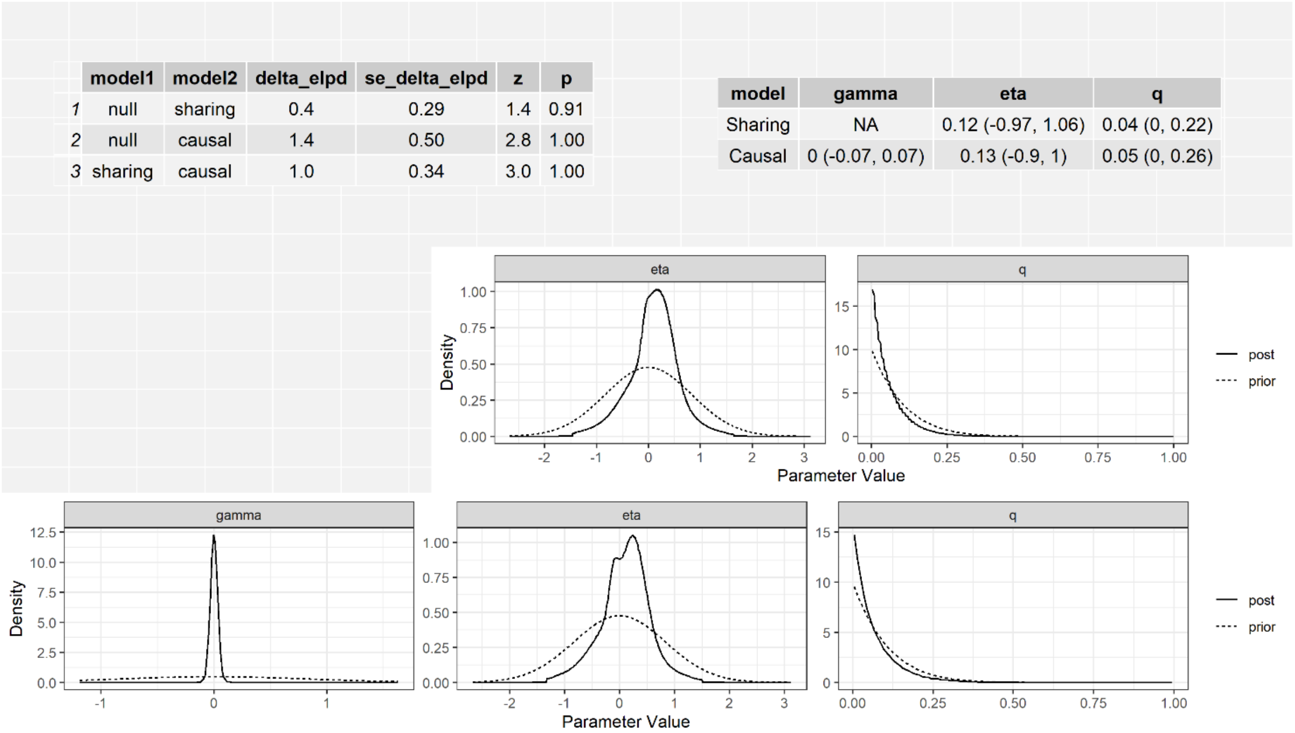

	model1	model2	delta_elpd	se_delta_elpd	z	p
1	null	sharing	0.4	0.29	1.4	0.91
2	null	causal	1.4	0.50	2.8	1.00
3	sharing	causal	1.0	0.34	3.0	1.00

model	gamma	eta	q
Sharing	NA	0.12 (-0.97, 1.06)	0.04 (0, 0.22)
Causal	0 (-0.07, 0.07)	0.13 (-0.9, 1)	0.05 (0, 0.26)

Supplementary Figure 10: Univariate MR analysis of atrial fibrillation on breast cancer, results of the CAUSE model.

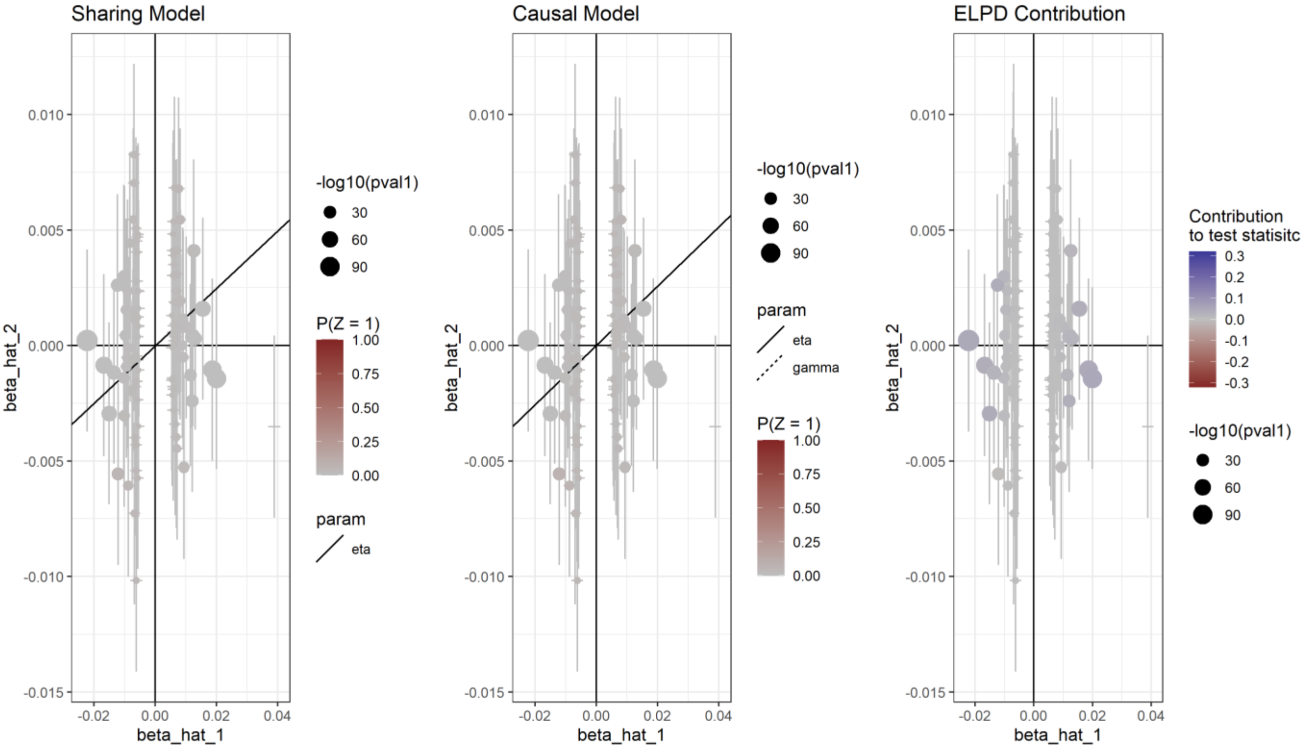

Supplementary Figure 11: Scatter plot of the CAUSE model results from the univariate MR analysis of atrial fibrillation on breast cancer.

	model1	model2	delta_elpd	se_delta_elpd	z	p
1	null	sharing	0.41	0.23	1.8	0.96
2	null	causal	1.30	0.29	4.6	1.00
3	sharing	causal	0.92	0.13	7.2	1.00

model	gamma	eta	q
Sharing	NA	-0.02 (-0.22, 0.2)	0.04 (0, 0.22)
Causal	0 (-0.01, 0.02)	-0.02 (-0.21, 0.18)	0.04 (0, 0.26)

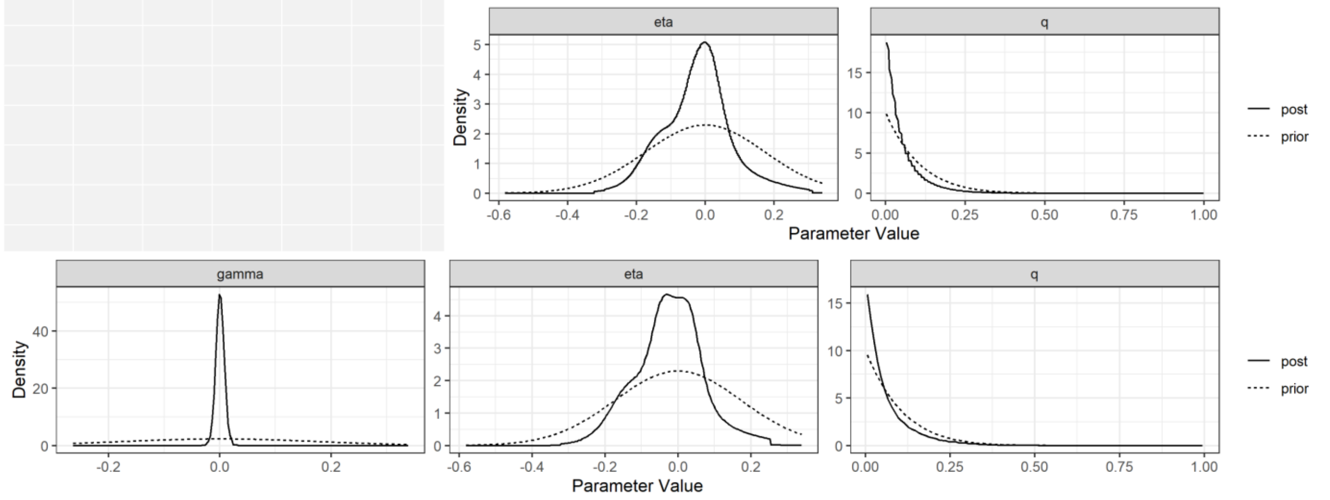

Supplementary Figure 12: Univariate MR analysis of breast cancer on atrial fibrillation, results of the CAUSE model.

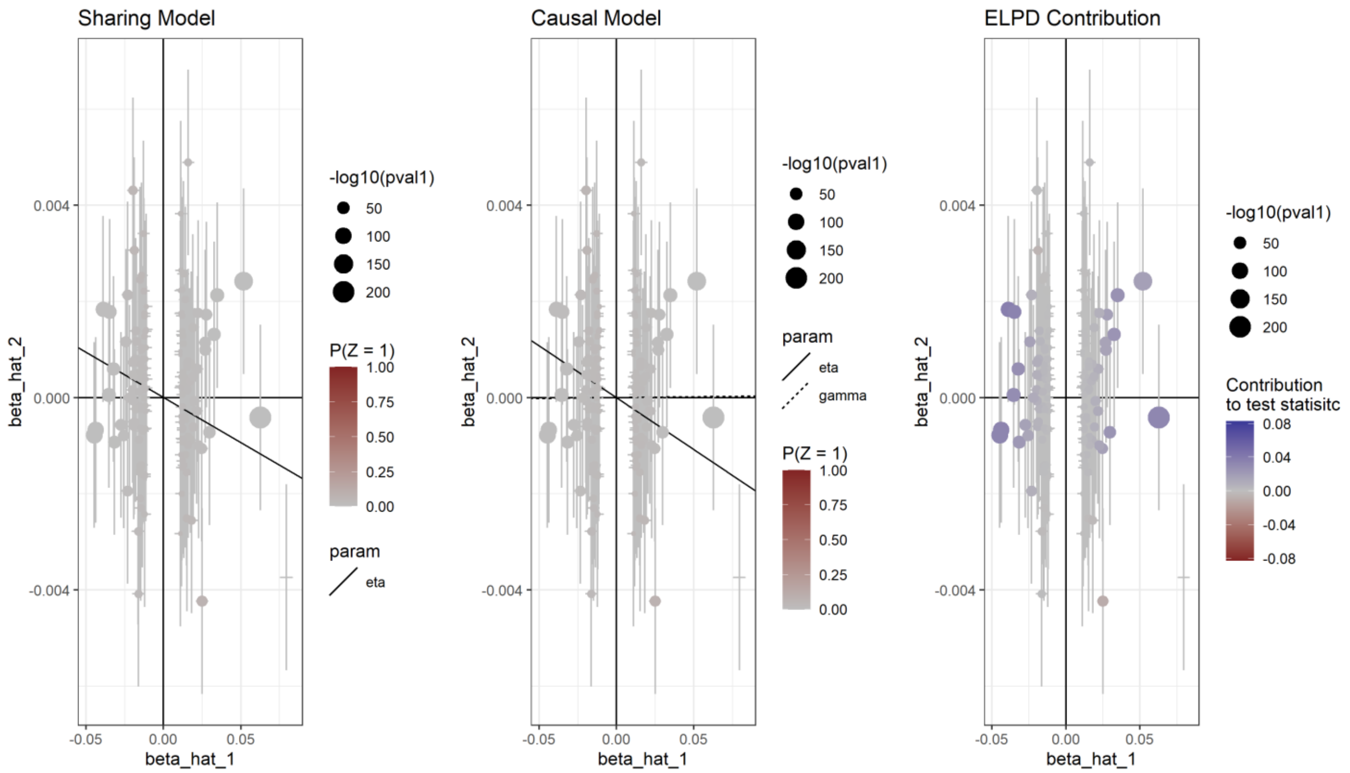

Supplementary Figure 13: Scatter plot of the CAUSE model results from the univariate MR analysis of breast cancer on atrial fibrillation.
